# Atlanta Medical College

**Published:** 1872-08

**Authors:** 


Atlanta Medical College.—We direct attention to the announcement of the Atlanta Medical College, to be found in the advertising department of thé Journal. As will be perceived, the new Faculty express strong hopes of a restoration of the old prosperity to the institution. We shall be pleased to know that they have realized their most sanguine anticipations in furnishing facilities for a thorough medical education, and in obtaining a full share of patronage.

We observe the election, among the new professors, of Dr. A. W. Calhoun (now in Vienna, Austria), one of the most promising men that the State of Georgia has produced; Dr. V. H. Taliaferro, of Columbus, Georgia, a distinguished gynæcologist; Dr. A. W. Griggs, of West-Point, Georgia, a man of talent and large and successful experience; and Dr. W. A. Love, of this city, well known to the readers of this journal as a man of progress and marked attainments in medical science.

